# Retrospective five-year study of equine casuistry in a Colombian perinatology center

**DOI:** 10.29374/2527-2179.bjvm005824

**Published:** 2025-05-30

**Authors:** Melissa Tovar Parra, Martha Susana Franco Ayala

**Affiliations:** 1 Universidad de los Andes, Bogotá, Colombia; 2 Universidad de la Salle, Bogotá, Colombia; 3 Universidad Nacional de Colombia, Programa de Doctorado en Salud Animal, Bogotá, Colombia

**Keywords:** foal, neonate, survival, pathologies, prognosis, potro, neonato, sobrevivência, patologias, prognóstico

## Abstract

A retrospective study was conducted at the Foal Care Equine Perinatology Center in Colombia from October 2017 to May 2023. The goal was to analyze the most frequent pathologies, as well as assess the sexes, ages, and breeds of the patients, with an emphasis on the perinatal population. Out of 945 clinical histories, 776 pertained to perinatology. Survival rates were calculated, and a descriptive analysis was performed. The overall survival rate for neonates was 73.6%, with females constituting 57% of the neonatal patients. The most prevalent breed among mares and neonates was the Creole Colombian Horse. Neonatal maladjustment accounted for 39.34% of the neonatal cases, and regarding the affected systems, there was a common multisystemic presentation (71.40%). Of the 341 foals admitted for neonatal adaptation, 72 were clones (with a survival rate of 76.39%), while 269 were non-clones (with a survival rate of 72.49%). A total of 202 mares (88.98%) were hospitalized for pregnancy monitoring, while 25 mares (11.02%) were brought to the perinatology center to treat dystocia or other reproductive pathologies. The most frequent diagnosis among mares was placentitis (73.68%). This study noted an increase in survival rates over time, which was associated with improvements in protocols and the acquisition of new medical equipment. The survival rates observed in this study are consistent with those reported in previous research. The collected data indicate that foaling occurs year-round in Colombia since the country experiences no distinct seasons. Studies with larger sample sizes are suggested, as well as periodical analysis of casuistry in the equine neonatology field, to improve the clinical management of patients and enhance productivity in the horse breeding industry.

## Resumo

Foi realizado um estudo retrospectivo no Centro de Perinatologia Equina Foal Care, na Colômbia, entre outubro de 2017 e maio de 2023. O objetivo foi analisar as patologias mais frequentes, bem como avaliar os sexos, idades e raças dos pacientes, com ênfase na população perinatal. Das 945 fichas clínicas avaliadas, 776 estavam relacionadas à perinatologia. As taxas de sobrevivência foram calculadas e realizou-se uma análise descritiva. A taxa geral de sobrevivência dos neonatos foi de 73,6%, sendo que as fêmeas representaram 57% dos pacientes neonatais. A raça mais prevalente entre éguas e neonatos foi o Cavalo Crioulo Colombiano. A síndrome do neonato desadaptado correspondeu a 39,34% dos casos neonatais, e quanto aos sistemas afetados, houve uma apresentação multissistêmica comum (71,40%). Dos 341 potros admitidos para adaptação neonatal, 72 eram clones (com taxa de sobrevivência de 76,39%), enquanto 269 não eram clones (com taxa de sobrevivência de 72,49%). Um total de 202 éguas (88,98%) foi hospitalizado para monitoramento gestacional, enquanto 25 éguas (11,02%) foram levadas ao centro de perinatologia para tratamento de distocia ou outras patologias reprodutivas. O diagnóstico mais frequente entre as éguas foi a placentite (73,68%). O estudo observou um aumento nas taxas de sobrevivência ao longo do tempo, associado à melhoria nos protocolos e à aquisição de novos equipamentos médicos. As taxas de sobrevivência observadas neste estudo são consistentes com as relatadas em pesquisas anteriores. Os dados coletados indicam que os partos ocorrem ao longo de todo o ano na Colômbia, uma vez que o país não apresenta estações definidas. Sugere-se a realização de estudos com amostras maiores, bem como análises periódicas da casuística em neonatologia equina, para aprimorar o manejo clínico dos pacientes e aumentar a produtividade na indústria da criação de cavalos.

## Introduction

The perinatal stage is a critical phase in equine breeding, encompassing pregnancy and the neonatal period. Several factors have been identified as contributing to abortion, stillbirths, and fetal mortality during this time. For instance, a study by Giles et al. (1993) found placentitis caused by bacteria or fungi and placental infections resulting from herpesvirus to be the most common causes of mortality in the perinatal period. These were followed by complications during birth, such as asphyxia and dystocia. Additionally, the study reported other frequent causes of perinatal death to include leptospirosis, improper placental separation, placental edema and insufficiency, placental villous atrophy, twin pregnancies, contracted foal syndrome, abnormalities of the umbilical cord, and congenital abnormalities.

These findings are consistent with those reported by Hong et al. (1993) from a study in Kentucky, USA. In their research, the authors identified placentitis and neonatal asphyxia, often related to dystocia, as the most common causes of perinatal death, followed by various infectious and non-infectious factors.

A recent study in Australia highlighted the most common causes of abortion-related pathologies such as equine amnionitis and fetal loss (EAFL). These causes include poor blood perfusion to the fetus, placentitis, and other infectious agents such as herpesviruses 1 and 4 (Carrick et al., 2014). Silva et al. (2020), meanwhile, found bacterial infections to be the leading cause of abortions, fetal losses, and perinatal deaths. The most frequently identified bacteria were *Escherichia coli*, *Enterobacter aerogenes*, *Streptococcus* spp., *Staphylococcus* spp*.,* and *Bacillus* spp. Other pathogens involved were *Leptospira spp*. and herpesviruses 1 and 4, as noted in previous studies.

A study performed in Brazil by Juffo et al. (2022) found that abortions and perinatal deaths were primarily caused by infectious pathologies such as placentitis, often linked to bacterial infections. In contrast, these authors observed that most stillbirths were caused by non-infectious factors, including dystocia and perinatal asphyxia.

The neonatal stage encompasses the period from day zero to day fifteen after a foal is born. This is a critical stage for equines as perinatal adaptation occurs, with the newborn quickly adjusting its body systems to function outside the uterus. Failure of this process can jeopardize the foal’s development and survival (Bazzano et al., 2014; Lyle‐Dugas et al., 2016).

Consequently, the neonatal stage is considered one of the highest-risk periods in the equine breeding industry. There are high mortality and morbidity rates during this stage, ranging from 0.38% to 22% and 25% to 88.8%, respectively (Franco Ayala & Oliver Espinosa, 2015). 

Vulnerability arises from newborn foals’ susceptibility to various health issues, including neonatal maladjustment syndrome, prematurity, dysmaturity, and neonatal septicemia. Other conditions can also pose serious risks, such as respiratory problems, diarrhea, fever, glucose dysregulation, musculoskeletal diseases, and trauma (Bazzano et al., 2014; Lyle‐Dugas et al., 2016).

A study on healthy equine breeding farms in Colombia revealed a neonatal survival rate of 99.23%, while the morbidity rate reported was 27.7% (Franco Ayala & Oliver Espinosa, 2015). However, there is an absence of similar studies focusing on hospitalized equine neonates in Colombia. In other countries, mortality rates reported in critically ill neonatal foals admitted to intensive care units range from 20% to 60%. These foals often suffer from conditions such as sepsis, prematurity, trauma at birth, and neonatal maladjustment syndrome, among others (Dembek et al., 2014; Giguère et al., 2015).

Survival rates for critically ill neonatal foals have been reported globally, with the most recent studies showing rates above 80%. For instance, the retrospective study by Bohlin et al. (2019) found a survival rate of 83%, based on data from 576 newborn foals admitted to five equine hospitals in Denmark and Sweden between 2007 and 2017. Similarly, Bedenice et al. (2021) reported a survival rate of 82% for 356 neonatal foals admitted to the Tufts Large Animal Hospital at the Cummings School of Veterinary Medicine from February 2001 to May 2016.

Previous studies indicated lower survival rates for neonatal foals. A retrospective study by Giguère et al. (2015) reported a survival rate of 72.8% among 1,065 neonatal foals admitted to the Large Animal Hospital at the University of Florida from 1982 to 2008. Lyle‐Dugas et al. (2016), meanwhile, reported a survival rate of 79.8% for 94 foals diagnosed with neonatal encephalopathy who were admitted to the same hospital between January 1996 and June 2007. Elsewhere, a study by Saulez et al. (2007) found a survival rate of 66% among 62 neonates hospitalized in intensive care units at a private hospital in South Africa between January and July 2004.

Studies in equine perinatology are essential for several reasons. Firstly, they enable clinicians to assess survival and mortality rates in equine hospitals. Additionally, these studies help identify disease characteristics, the prevalence of various pathologies, and the most affected systems within a specific population. Collectively, this information is crucial in enhancing the prognosis for hospitalized equine neonates (Pantaleon, 2019).

Furthermore, such studies allow veterinarians to identify clinical variables associated with non-survival and to establish appropriate treatment and management protocols for patients. Recognizing these clinical variables is extremely important, as clinical examinations often have limited predictive power regarding the prognosis and outcomes in critically ill foals (Hoffman et al., 1992).

Predicting outcomes in equine veterinary care is both important and challenging. Given the high costs associated with hospitalization and treatments, accurate predictions at the time of admission are crucial. These facilitate clearer communication between veterinarians and horse owners regarding the likelihood of survival, as well as the horse’s potential for sports performance, among other factors (Bohlin et al., 2019; Castagnetti & Veronesi, 2008; Rohrbach et al., 2006; Wilkins, 2015).

Accordingly, the primary aim of the present study was to analyze the most common diagnoses and affected systems at an equine perinatology center in Colombia. It also sought to determine the sexes, ages, and breeds of the patients, as well as their survival rates and mortality. Special attention was given to the population in the perinatal stage.

## Materials and methods

A retrospective analysis was conducted from May 2017 to October 2023 at the Foal Care Equine Perinatology Center, Cundinamarca, Colombia. The location has a latitude of 4.917°N (4°55’06’’N), longitude of -74.017°W (74°01’40’’W), height of 2,559 meters above sea level, and average temperature of 14 °C.

A total of 945 medical records were analyzed, including 776 perinatal clinical cases. The data collected from each medical record included breed, sex, age, working diagnosis, affected system, and the outcome after hospitalization (survived, deceased, or euthanized).

The following breeds were represented: Colombian Creole Horse, jumping breeds (including Hanoverian, Holstein, Warmblood, French Saddle, Argentine Saddle, Colombian Sport Horse, and Trakehner, among others), Argentine Polo, Mini Horse, Pony, Friesian, Belgian, Percheron, Spanish Purebred, Thoroughbred, Lusitano, Quarter Horse, Mini Donkey, Gypsy Vanner, Donkey, Baroque Pinto, American Pinto, Andalusian Horse, Mini Gypsy, Mammoth Donkey, Appaloosa, and Mule.

The age categories were as follows: neonate from 0 to 30 days, foal from 30 days to 1-year, young horse from 1 to 4 years, mares that entered the clinic for pregnancy monitoring or pathologies related to pregnancy, and adults at over 4 years old (both mares and other horses).

The working diagnosis was established as the most likely pathology within the differential diagnoses. Depending on this pathology and its effects, the patient was assigned to a category of system affected, such as multisystemic, reproductive, gastrointestinal, musculoskeletal, respiratory, nervous, immune, or cardiovascular. Finally, the potential outcomes were analyzed.

Descriptive statistical analysis was performed using Microsoft Office Excel 365 ProPlus^®^ software. A comprehensive database was created for frequency analysis of each variable, with an emphasis on perinatology. Neonate and mare mortality and survival rates were calculated for each year, along with overall survival rates for the entire population (neonates, foals, young horses, and adults). Percentage analysis was conducted of the final diagnoses and systems affected. Finally, this study recorded the number of clones treated, along with their survival and mortality rates.

## Results

Between May 2017 and October 2023, 945 equine patients were hospitalized at the Foal Care Perinatology Center, comprising 64.97% (n=614) females and 35.03% (n=331) males.

**Figure 1:**
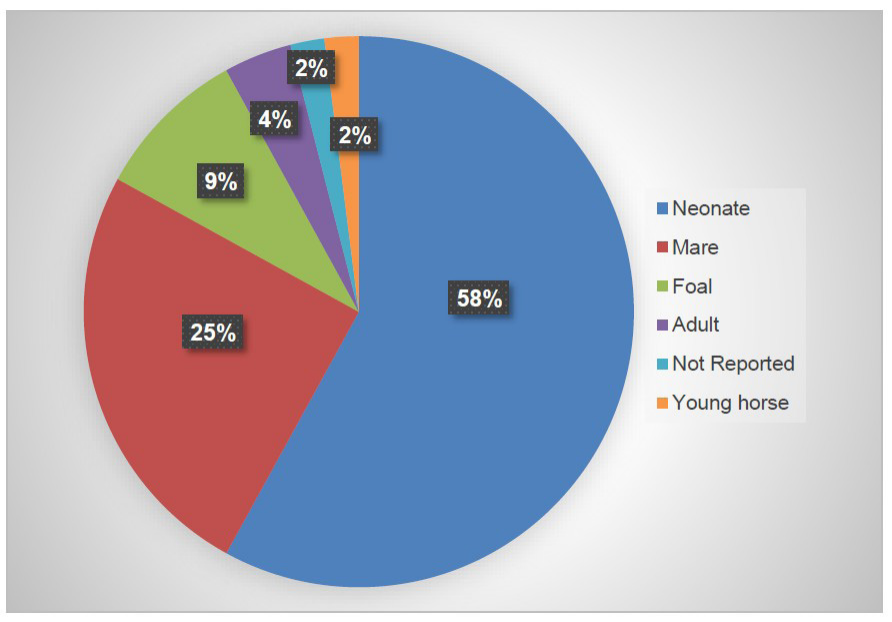
Distribution of Patients According to Age.

Note. Distribution of patients according to age category between October 2017 and May 2023 is shown in a pie chart. Th most representative age category was neonate foal, followed by mares.

The majority were neonates, accounting for 58.1% (n=549) of the cases, followed by mares, who made up 24.02% (n=227). Only 9% (n=80) of the cases involved colts and fillies (foals) aged between 30 days and one year old. Just 4% (n=40) were classified as adults, which included horses with various diseases and mares with non-reproductive conditions. Young horses aged one to four years constituted 2% (n=20) of the cases, and in another 2% (n=20) of the cases, the age was not reported (see Figure 1).

**Figure 2:**
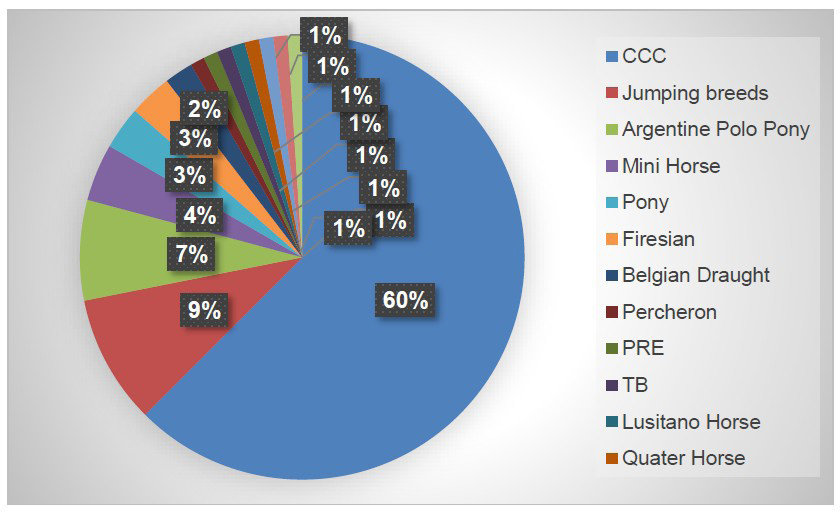
Distribution of Patients According to Race.

Note. Distribution of patients according to breed between October 2017 and May 2023 is shown in a pie chart. The most representative breed was creole Colombian horse (CCC), followed by jumping category.

Most patients (60.0%) belonged to the Colombian Creole Horse breed. Other breeds included jumping breeds (8%), Argentine Polo horses (7%), Mini Horses (4%), and Ponies (4%). Friesians made up 3%, while Belgians constituted 2%. Breeds such as Percheron, Pura Raza Español, Thoroughbred, Lusitano, Quarter Horse, Mini Donkey, Gypsy Vanner, and Donkey each represented 1% of the population, while breeds such as Baroque Pinto, American Pinto, Andalusian, Mini Gypsy, Mammoth Donkey, Appaloosa, and Mule accounted for less than 1% each. In 4% of cases, the breed was unreported (NR) (see Figure 2).

Neonatal maladjustment syndrome was the most frequent diagnosis (22.85%, n=147), followed by high-risk pregnancy, which represented 21.90% of cases (n=207). The mare category included several conditions that decreased the viability of the foal and increased the risk of developing pathologies such as placentitis (n=16), placental insufficiency (n=6), and hydroallantois (n=1), along with other related conditions.

Among the categories of systems affected, the multisystemic category was the most highly represented, at 42.22% (n=399) of the clinical cases. This was followed by the reproductive system, which comprised 25.50% (n=241/945), and the gastrointestinal system, at 11.64% (n=110/945). The musculoskeletal and respiratory systems had similar case numbers, representing 6.14% (n=58/945) and 5.61% (n=53/945) of the studied population, respectively.

The survival rate for the five-year sample at the Perinatology Center was 78.3% (n=740) and the mortality rate was 21.69% (n=205), with 3% of those cases euthanized. During the study period, both the number of cases and the survival rate increased, with notable peaks of both in 2020.

A more detailed analysis of the perinatal stage yielded the following results: Of the 945 cases, 776 were perinatal. Among those, 549 cases involved neonatal foals, while 227 cases pertained to mares with reproductive issues related to pregnancy and parturition.

**Figure 3:**
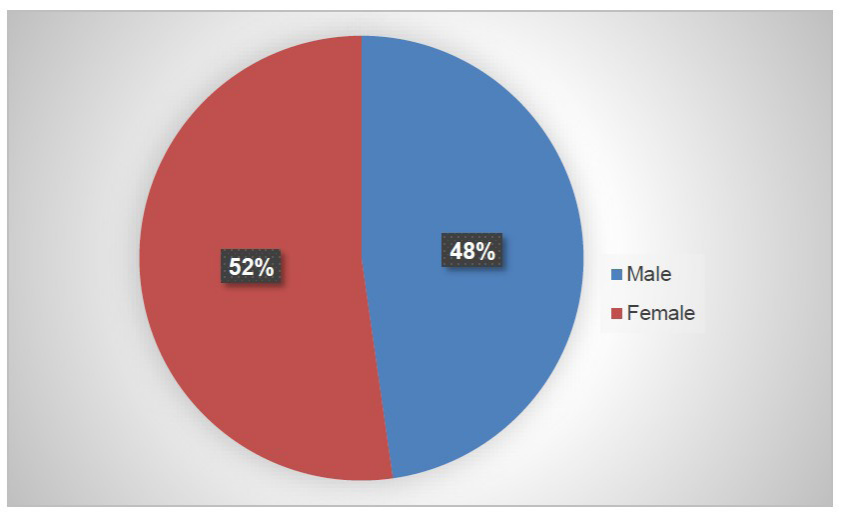
Distribution of Neonatal Patients by Sex.

**Note.** Distribution of neonatal patients according to sex shown in a pie chart. There is an even Distribution of males and females.

**Figure 4:**
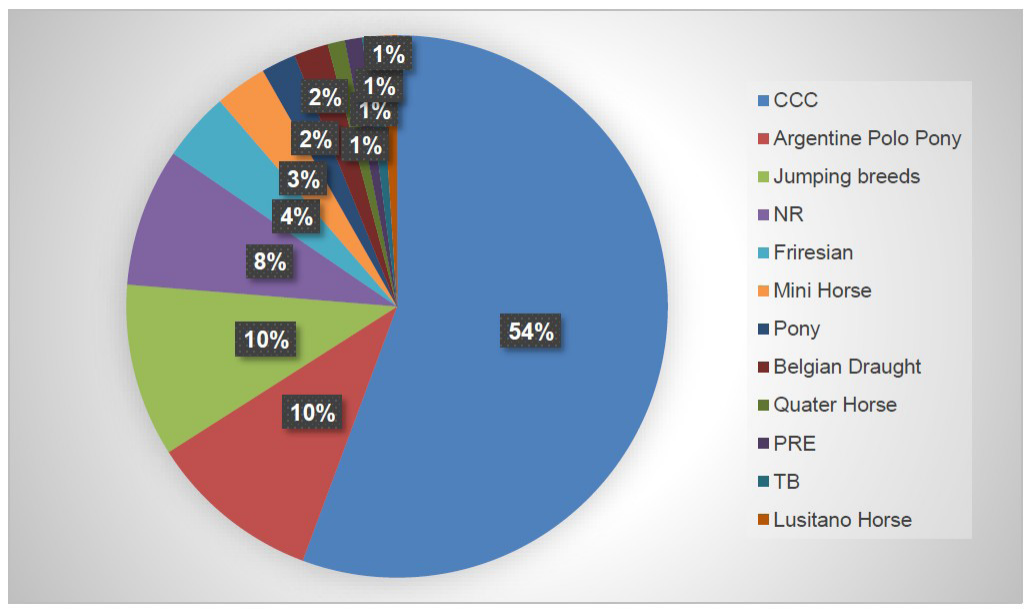
Distribution of Neonatal Patients According to Race.

**Note.** Distribution of neonatal patients according to breed shown in a pie chart. The most representative breed was creole Colombian horse (CCC), followed by jumping category.

Among the neonatal foals, 52.23% (n=287) were female and 47.72% (n=262) were male (see Figure 3). The most common breed in the neonatal group was the Colombian Creole Horse (54%). This was followed by the Argentine Polo Pony and jumping breeds, each representing 10% of the population. In 8% of the cases, the breed was not reported (NR). Additionally, 4% of the neonatal foals were Friesian, 3% were Mini Horses, 2% were Ponies, and 2% were Belgian Draughts. The remaining breeds made up 1% or less of the neonatal population (see Figure 4).

**Figure 5:**
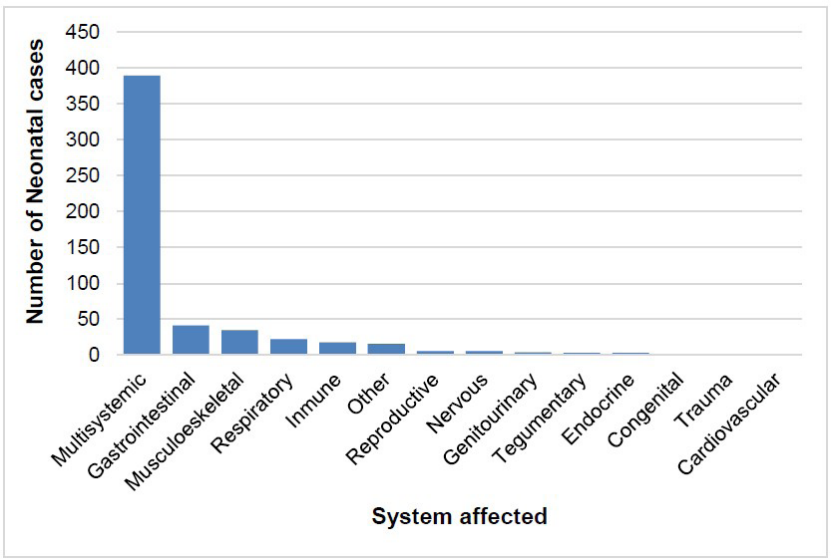
Distribution of Neonatal Patients by Affected System.

Note. Distribution of cases in the neonatal population according to affected system is shown. The figure includes data between October 2017 and May 2023.

Most neonates had multisystemic effects, observed in 71% (n=389) of the cases. This was followed by gastrointestinal issues in 9% (n=41) and musculoskeletal problems in 7% (n=35) of the neonatal patients. Other systems were affected in 4% or fewer neonatal foals (see Figure 5 and Table 1).

**Table 1:** Classification of Neonatal Cases According to Diagnosis and System Affected.

**Body System**	**Diagnosis**	**n**	**%**
**Multisystemic**	Neonatal Maladjustment	216	39.34
	Neonatal Septicemia	79	14.39
	Foal prematurity	52	9.47
	Neonatal immaturity	42	7.65
	Tetanus	3	0.55
	**Total**	**391**	**71.40%**
**Gastrointestinal**	Diarrhea	18	3.28
	Meconium impaction	11	2
	Acute abdominal syndrome	7	1.27
	Enterocolitis	5	0.91
	Enteritis	2	0.36
	Colitis	2	0.36
	Esophageal obstruction	1	0.18
	Gastric obstruction	1	0.18
	Atresia coli	1	0.18
	Intussusception	1	0.18
	**Total**	**49**	**8.93%**
**Musculoskeletal**	Septic arthritis	23	4.19
	Head trauma	4	0.73
	Fracture	3	0.55
	Angular limb deformities	3	0.55
	Trauma	3	0.55
	Fracture	3	0.55
	Tendinitis	1	0.18
	Partial agenesis of the atlas	1	0.18
	Necrotizing fasciitis	1	0.18
	**Total**	**42**	**7.65%**
**Respiratory**	Pneumonia	10	1.82
	Respiratory distress	5	0.91
	Meconium aspiration	3	0.55
	Pleuropneumonia	3	0.55
	Equine adenitis	1	0.18
	**Total**	**22**	**4.00%**
**Nervous**	Neurological signs	1	0.18
	Seizures	1	0.18
	**Total**	**22**	**0.36%**
**Immune**	Failure of passive transfer (FPT)	17	3.1
	Neonatal isoerythrolysis	1	0.18
	**Total**	**18**	**3.78%**
**Cardiovascular**	Endocarditis	1	0.18
	**Total**	**1**	**0.18%**
**Genitourinary**	Uroperitoneum	2	0.36
	Umbilical hernia	1	0.18
	Omphalitis	1	0.18
	**Total**	**4**	**0.73%**
**Integumentary**	Mastitis	1	0.18
	**Total**	**1**	**0.18%**
**Endocrine**	Hypoglycemia	2	0.36
	**Total**	**2**	**0.36%**
**Reproductive**	Agalactia	5	0.91
	Scrotal Hernia	1	0.18
	**Total**	**6**	**1.10%**
**Other**	Orphan	13	2.37
	**Total**	**15**	**1.59%**
**TOTAL**		**549**	**100%**

Note. Each diagnosis was assigned to an organ system and the number of neonatal cases per diagnosis was recorded. The last row shows what percentages of the total population were hospitalized for a specific diagnosis.

As shown in Table 1, the most frequent diagnosis among these foals was neonatal maladjustment syndrome affecting 39.16% (n=215) of the cases. That was followed by neonatal septicemia in 14.39% (n=79), prematurity in 9.47% (n=52), and immaturity in 7.65% (n=42) of the neonates. Additionally, 4.2% (n=23) of the foals were hospitalized due to septic arthritis, 3.1% (n=17) experienced failure of passive transfer, and 3.3% (n=18) had diarrhea. Other diagnoses accounted for 2% or fewer of the neonates, as detailed in Table 2.

**Table 2:** Survival of Neonatal Cases According to Diagnosis.

**Working diagnosis**	**Lives**	**Dies**	**Euthanasia**	**Total**	**Survival Rate**	**Mortality Rate**
	n	n	n	n	%	%
Maladjustment syndrome	167	42	6	215	77.67	22.33
Neonatal septicemia	48	28	3	79	60.76	39.24
Prematurity	29	18	5	52	55.77	44.23
Immaturity	25	14	3	42	59.52	40.48
Septic arthritis	21	1	1	23	91.30	8.70
Failure of passive transfer	17	2	0	17	100	11.76
Diarrhea	18	0	0	18	100	0
Orphan	13	0	0	13	100	0
Meconium impaction	11	0	0	11	100	0
Acute abdominal syndrome	5	2	0	7	71.43	28.57
Pneumonia	7	3	0	10	70	30
Enterocolitis	2	3	0	5	40	60
Respiratory distress	2	3	0	5	40	60
Agalactia	3	2	0	5	60	40
Head trauma	3	1	0	4	75	25
Tetanus	0	2	1	3	0	100
Angular limb deformities	2	1	0	3	66.67	33.33
Meconium aspiration	3	0	0	3	100	0
Fracture	2	1	0	3	66.67	33.33
Trauma	3	0	0	3	100	0
Enteritis	0	0	0	2	0	0
Colitis	2	0	0	2	100	0
Hypoglycemia	2	0	0	2	100	0
Pleuropneumonia	3	0	0	3	100	0
Atresia Coli	1	0	0	1	100	0
Scrotal hernia	1	0	0	1	100	0
Tendinitis	1	0	0	1	100	0
Mastitis	1	0	0	1	100	0
Umbilical hernia	1	0	0	1	100	0
Necrotizing fasciitis	1	0	0	1	100	0
Seizures	1	0	0	1	100	0
Neonatal isoeritrolisis	1	0	0	1	100	0
Uroperitoneum	2	0	0	2	100	0
Esophageal obstruction	1	0	0	1	100	0
Lameness	1	0	0	1	100	0
Endocarditis	0	1	0	1	0	100
Omphalitis	0	1	0	1	0	100
Gastric obstruction	1	0	0	1	100	0
Intussusception	1	0	0	1	100	0
Equine Adenitis	1	0	0	1	100	0
Partial agenesis of the atlas	1	0	0	1	100	0
Neurologic	0	1	0	1	0	100
**Total**	404	126	19	549		
		145				

**Note:** For each diagnosis the outcome, number of total cases, percentages over total neonatal population, as well as survival and mortality rates are shown in the table.

The overall survival rate for the neonatal foals was 73.59%, with a mortality rate of 26.41%. In total, 145 foals died, with euthanasia performed in 19 of those cases. Specific survival and mortality rates for each diagnosis are presented in Table 2.

Foals diagnosed with maladjustment syndrome had a survival rate of 77.67% (n=167 out of 215) and a mortality rate of 22.33% (n=48 out of 215), with five of those foals euthanized. Meanwhile, foals with neonatal septicemia showed a survival rate of 60.76% (n=48 out of 79) and a mortality rate of 39.24% (n=31 out of 79); in three of those foals, euthanasia was applied (see Table 2).

In the multisystem category, 341 neonatal foals underwent perinatal adaptation. This group included those diagnosed with conditions such as neonatal maladjustment, prematurity, dysmaturity, and passive transfer failure and those orphaned. The patients were divided into two categories: clones, which comprised 13.14% of the total neonatal population (n=72), and non-clones, making up 49.09% (n=269). Among the 72 clones, the survival rate was 76.39% (n=55), while the non-survival rate was 23.61% (n=17), including four patients that were euthanized. In the non-clone category, the survival rate was 72.49% (n=195), with a non-survival rate of 27.50% (n=74), which included 10 patients euthanized.

**Figure 6:**
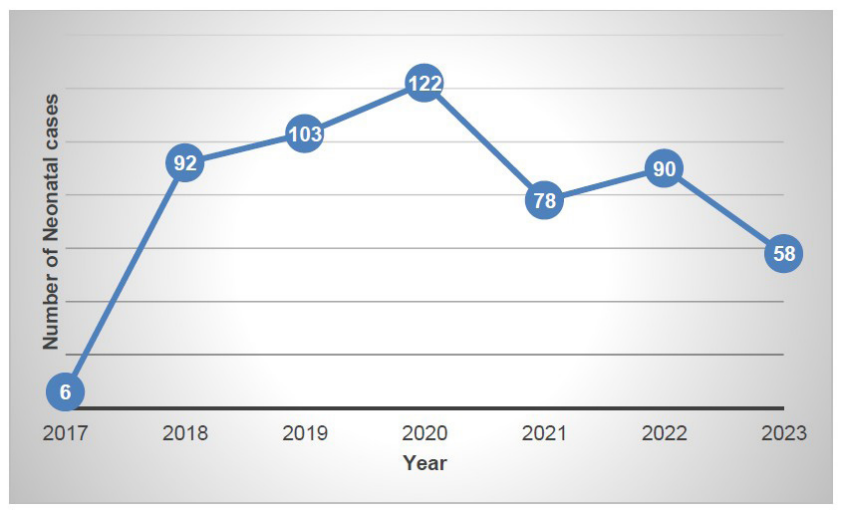
Number of Neonatal Cases in each Year of Clinic Functioning.

**Note.** Total number of neonatal cases in each of the years of operation of the equine perinatology center.

**Figure 7:**
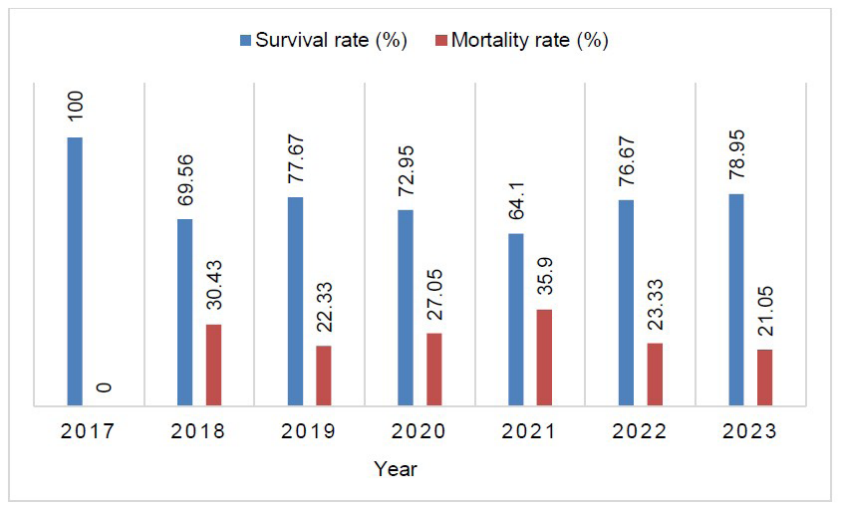
Survival and Mortality rates in the neonatal population over time.

**Note.** Percentages of survival and mortality are shown for each year of functioning of the perinatology center for the neonatal population.

During the five years under study, neonatal cases increased from 2018 to 2020, followed by a decrease from 2021 to 2023. The survival rates during this period ranged from 64.1% to 78.95%, with rates exceeding 70% in most years. The highest survival rate was recorded in 2017 and 2023. Details of the number of cases per year, along with survival and mortality rates, are given in Figures 6 and 7.

**Figure 8:**
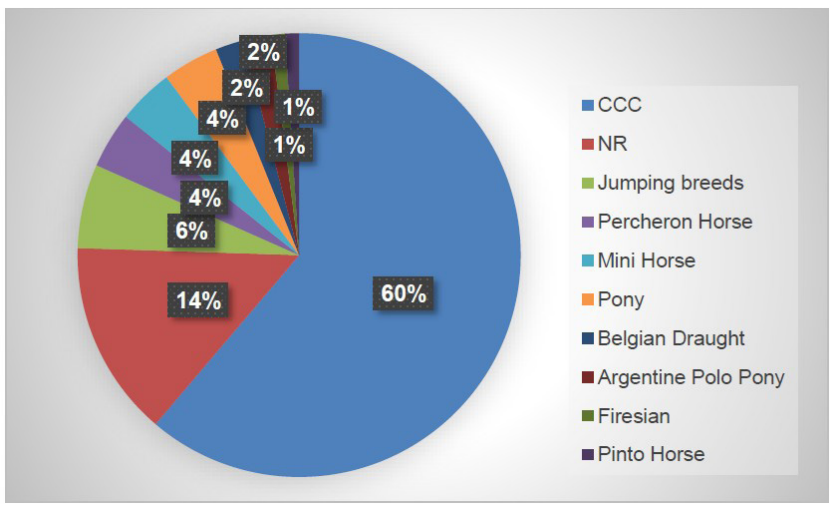
Distribution of Mares According to Race.

**Note.** Distribution of mares according to breed shown in a pie chart. The most representative breed was Creole Colombian horse (CCC).

In the group of mares, the most represented breed was the Colombian Creole Horse, which comprised 60% (n=137) of the population. For 14% of the mares, the breed was not reported. Meanwhile, 6% of the mares were jumping breeds (n=14), and the Percheron horse made up 6% (n=10) of the group. Other breeds each represented 4% or less of the assessed population (see Figure 8).

All mares were categorized based on their reproductive status. A total of 202 mares (88.98%) were hospitalized for pregnancy monitoring, and 25 mares (11.02%) were taken to the perinatology center for the treatment of dystocia (n=14) or postpartum pathologies, which included metritis (n=2), agalactia (n=3), periparturient hemorrhage (n=4), and retained fetal membranes (n=2).

**Figure 9:**
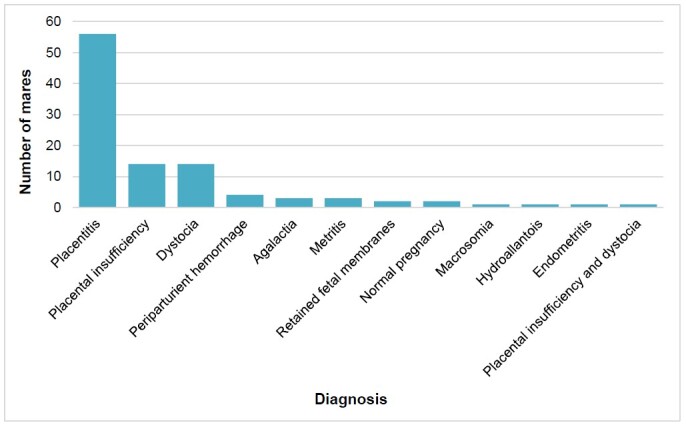
Frequent Diagnoses in Mares Admitted for Pregnancy Monitoring and Postpartum Care.

**Note.** The graph shows the Distribution of mares according to the working diagnosis. In 76 mares that were admitted for pregnancy monitoring the diagnosis was reported, the Distribution is shown in this graph.

Of the 202 mares hospitalized for pregnancy monitoring, a final diagnosis was reported for 76 of them. The most common diagnosis was placentitis, occurring in 73.68% (n=56) of cases, followed by placental insufficiency, at 18.42% (n=14). There was one case of metritis (1.32%), one mare presented macrosomia (1.32%), and another mare presented hydroallantois (1.32%). Two mares experienced normal pregnancies (2.63%) but were admitted for monitoring. Additionally, one mare developed endometritis (1.32%). One mare with placental insufficiency experienced dystocia during delivery and required a cesarean section (see Figure 9).

**Figure 10:**
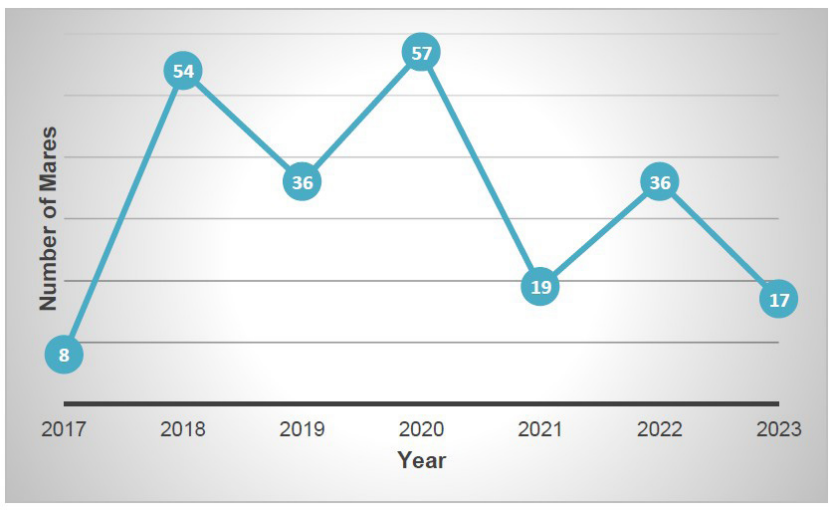
Number of Mares Hospitalized over time.

**Note.** Total number of mares in each of the years of operation of the equine perinatology center.

**Figure 11:**
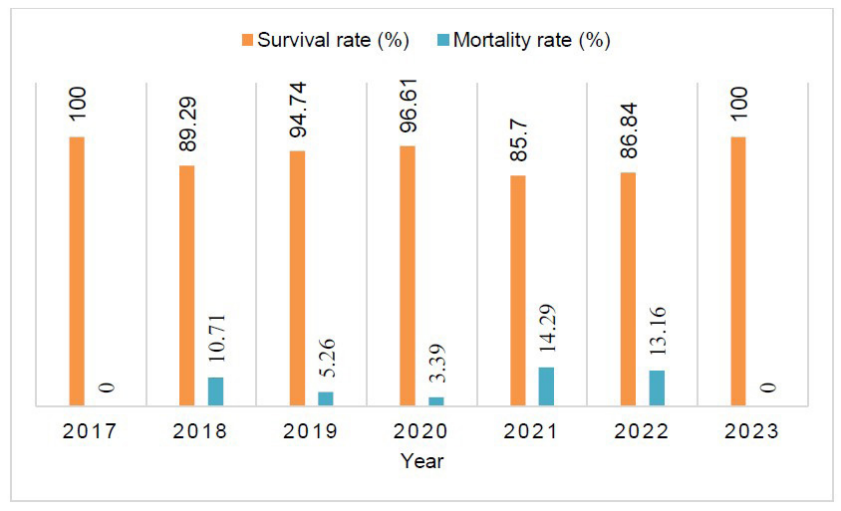
Survival and Mortality rates in Mares over time.

**Note.** Percentages of survival and mortality are shown for each year of functioning of the perinatology center for the mares’ population.

The survival rate among mares was 93.83% (n=213), with a mortality rate of 6.17% (n=14). One of the deaths resulted from euthanasia, accounting for 0.44% of the mares. The number of mares hospitalized in the perinatology center increased between 2017 and 2020; however, there was a significant decline in 2021 and 2022, as illustrated in Figure 10. Survival rates varied between 85.7% and 100%. Detailed information regarding the number of cases per year, along with survival and mortality rates, can be found in Figures 10 and 11.

**Figure 12:**
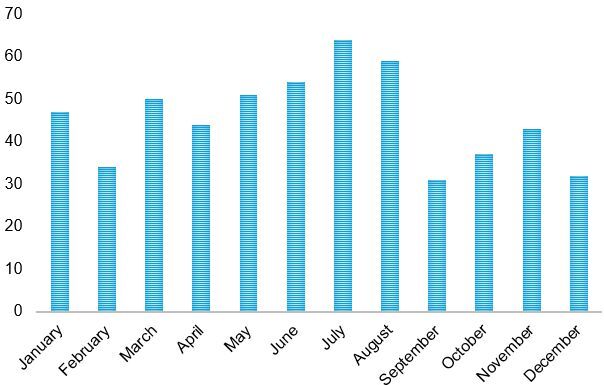
Number of Foals Hospitalized each Month.

**Note.** Neonatal foals are hospitalized during all months of the year.

**Figure 13:**
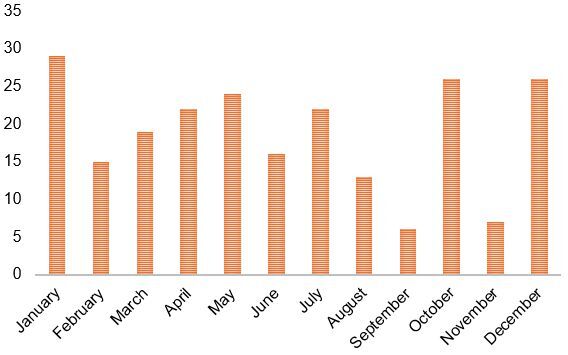
Number of Mares Hospitalized each Month.

**Note.** Mares are hospitalized during all months of the year for pregnancy monitoring.

It was observed that neither the number of foals nor the number of mares hospitalized correlated with the month of the year. The distribution of cases remained fairly consistent throughout the year, as depicted in Figures 12 and 13.

## Discussion

This study represents the first casuistry analysis at an equine perinatology center in Colombia, focusing on critically ill neonatal foals and pregnant mares. Previous epidemiological research primarily analyzed case, survival, and mortality rates among adult horses and did not specifically address neonatal foals or pregnant mares (Franco Ayala & Oliver Espinosa, 2015; Ujueta Rodríguez, 2019). As perinatal adaptation makes the neonatal period one of the highest-risk phases in equine production, it is essential to identify the diseases that affect both mares and neonatal foals during this vulnerable time (Bazzano et al., 2014; Daza Medina & Franco Ayala, 2023; Daza Medina et al., 2023), as an unsuccessful perinatal adaptation process jeopardizes the survival of foals.

Equine neonates are particularly vulnerable to conditions such as sepsis, neonatal maladjustment syndrome, prematurity, and other postpartum disorders, which can lead to high mortality rates (Dembek et al., 2014). Understanding their onset requires analyzing the characteristics of affected foals, the specific pathologies, the systems involved, and the outcomes of these conditions. It is crucial to gain knowledge in these ways as the medical management of critically ill neonatal foals is both expensive and demanding. In that context, predicting the chances of survival using clinical information and understanding the prognosis can aid veterinarians and horse owners in making informed decisions (Bohlin et al., 2019; Cruz et al., 2017; Dembek et al., 2014).

In accordance with the specialty of the clinic studied in this case, most patients were female (64.97%, n=614) and the most common age category was neonatal foals (58%, n=548), followed by mares hospitalized for pregnancy monitoring or other pregnancy-related pathologies (25%, n=227) (see Figure 1).

In Colombia, equine breeding occurs year-round due to the country’s equatorial climate, which lacks distinct seasons (Franco & Oliver-Espinosa, 2021). As a result, the number of hospitalized newborns and pregnant mares remains relatively constant throughout the year, as illustrated in this study (see Figures 12 and 13).

Most patients belonged to the Colombian Criollo Horse breed; that was true in the overall population (60%, n=567 out of 945), the neonatal foal category (54.09%, n=297 out of 549), and the mare category (60%, n=137 out of 227), as shown in Figures 2, 4, and 8. This breed is representative of the country. However, it was observed that a greater variety of breeds has been treated at the clinic over time due to its growth as a reference center and the recognition it has gained over the years.

The most common pathology observed was neonatal maladjustment syndrome, accounting for 22.85% (n=147) of cases. This aligns with the specialty of the Perinatology Center and differs from findings in other Colombian studies that focused on young and adult horses but not on foals. In those studies, the most frequently treated conditions related to the musculoskeletal system, affecting 43.8% of patients in one study (Ujueta Rodríguez, 2019) and 60.9% of horses in another (Franco Ayala & Oliver Espinosa, 2015). Additionally, pathologies of the hematopoietic system were reported in 37.6% of a hospitalized adult equine population (Cardona et al., 2017).

In the neonatal population specifically, the most frequent diagnosis was neonatal maladjustment syndrome, accounting for 39.16% of the neonatal foals, which was followed by neonatal septicemia in 14.39% of the neonatal foals (Table 1). It has been reported that these two conditions are the most frequent causes of morbidity and mortality in neonatal foals (Cruz et al., 2017). Therefore, diagnosing and appropriately and rapidly managing affected patients are greatly important.

The present study found a survival rate of 73.59% for neonatal foals, which is comparable to rates reported in previous research. For instance, Borchers et al. (2012) found a survival rate of 79% for 643 neonatal foals treated in university and private hospitals in 2008 and 2009. Similarly, Dembek et al. (2014) reported survival rates of 79% in a retrospective study (n=339) and 76% in a prospective study (n=285). Giguère et al. (2015), meanwhile, reported a survival rate of 72.8% in a study involving 1,065 critically ill neonatal foals from 1982 to 2008. Other studies have recorded higher survival rates; for example, Bedenice et al. (2021) reported an 82% survival rate in hospitalized neonatal foals, while Bohlin et al. (2019) noted an 83% survival rate in their study of 576 foals.

The survival rate for foals diagnosed with neonatal maladjustment syndrome in this study was 77.67% (see Table 2), consistent with the survival rate of approximately 80% reported by McKenzie III (2018) for similar cases. Interestingly, however, in this study, foals with neonatal sepsis had a survival rate of 60.76%, which was higher than expected based on the range of 26% to 57% reported also by McKenzie III (2018). Furthermore, for premature and immature foals, the survival rates were 55.77% and 59.52%, respectively (see Table 2), which were lower than the 80% to 85% survival rates noted in previous research (McKenzie III, 2018). Given the discrepancies that emerged from previous research, further investigation into these conditions seems warranted; future studies may increase the sample size for more accurate results. Additionally, studies that evaluate the treatment protocols for premature and immature foals may produce findings that help improve their survival rates.

The second most represented pathology in the perinatal population was high-risk pregnancy, at 21.90% (n=200), which included placentitis, placental insufficiency, hydroallantois, and other conditions that endangered the lives of the foal and the pregnant mare.

Further analysis of the mare category revealed that placentitis was the most frequent diagnosis in high-risk pregnancies, followed by placental insufficiency, while 11.02% of the hospitalized mares presented dystocia (see Figure 9); these results align with the most frequent causes of perinatal complications, such as abortions, fetal losses, and perinatal death, as reported by various authors (Carrick et al., 2014; Giles et al., 1993; Hong et al., 1993; Juffo et al., 2022; Silva et al., 2020).

The survival rate for mares was 96.62%. In comparison, the literature reports lower survival rates in specific cases: after a cesarean section, the survival rate reported ranges from 82% to 86%; after a fetotomy, it ranges from 71% to 91%; and after assisted delivery, it stands at 91% (Ellerbrock & Wehrend, 2023). The present study did not correlate the mare’s diagnosis with survival or assess the survival of the neonatal foals birthed by these mares. Further research correlating these medical conditions with the outcomes for the foals would be beneficial in understanding the impact of these diseases on foals in Colombia.

The increase in survival rates of critically ill neonatal foals is largely a result of studies that identify key parameters, indicators, and the prevalence of neonatal diseases, along with their associated morbidity and mortality rates. This research enables a detailed examination of the key parameters as risk factors for mortality, which in turn helps improve protocols related to diagnosis, treatment, and prognosis. These improvements advance clinical management and preventive practices (Abraham & Bauquier, 2021; Franco & Oliver-Espinosa, 2021; Graßl et al., 2017).

Continued advancements in reproductive and neonatal medicine are crucial for enhancing foal survival by reducing losses during pregnancy. This is achieved through ongoing improvements in the clinical management of high-risk pregnancies and the care of critically ill neonates (Abraham & Bauquier, 2021; Cruz et al., 2017).

Between 2018 and 2023, the survival rate of neonatal foals increased from 69.56% to 78.95% (see Figure 10). This improvement may be linked to enhanced care protocols and medical equipment for diagnosing and treating patients. It is crucial to identify the details around these factors, which could be explored in future case studies. Ongoing efforts aimed at evaluating clinical performance, standardizing care protocols, and advancing medical practices in equine neonatal and reproductive medicine in Colombia are all essential steps in this process.

In terms of annual case numbers, 2020 saw the highest caseloads among neonatal and mare populations. Conversely, the years 2017 and 2023 registered the lowest caseloads across all assessed populations (see Figures 6 and 10); however, the data for 2017 only covered the months from October to December, while the data for 2023 included only January through May.

The survival rate was highest in 2017, with an overall percentage of 89.47% for the entire population and a remarkable 100% for neonatal foals and mares. However, it is important to consider that, due to the limited sample size for 2017, the results for this year are not directly comparable to those of other years (see Figures 7 and 11).

An important area of further research in the present study is whether the use of cloning increases the risk of morbidity and mortality. Some studies suggest that there may be no significant difference, or even that there might be advantages in producing foals through nuclear transfer (Cortez et al., 2023). However, other research indicates potential complications, including early embryonic deaths, abortions, the birth of excessively large fetuses or placental abnormalities, premature foals, and weaker foals. As a result, gestation for cloned embryos is often considered a high-risk pregnancy that requires special attention (Chavatte-Palmer et al., 2014; Johnson & Hinrichs, 2015; Johnson et al., 2010).

A study on cloned equine neonates in Colombia revealed a higher number of deaths among cloned foals derived from fibroblastic cells compared to those from medullar cells and non-cloned foals. The most prevalent condition in fibroblastic-cell-origin clones was neonatal septicemia, which was linked to an increased mortality risk (odds ratio of 4.4) compared to non-cloned foals. This increase was likely associated with the development of placentitis in the mares carrying those foals (Franco & Oliver, 2024).

In the present study, the survival rate for cloned foals was 76.39% (n=55), while for non-cloned foals, it was 72.49% (n=195). However, the sample size presents a limitation, meaning this difference may not be statistically significant. Therefore, further research is necessary to explore this variable, using a larger sample size and a more detailed examination of the characteristics of these specific pregnancies.

An important recommendation for the future is to evaluate cases of death by euthanasia, which is often performed for financial reasons and because of poor prognoses related to quality of life or athletic performance. Additionally, it is crucial to assess the factors linked to natural deaths (Giguère et al., 2015). Euthanasia accounted for 3.46% of neonatal deaths in the present study, and future investigations should establish exclusion criteria based on the reasons behind the decision to perform euthanasia.

The limitations of this study stem from its retrospective nature; it relied solely on data collected from a single perinatology center, which may have introduced biases. Moreover, this resulted in incomplete information due to the reliance on clinical records. Furthermore, the sampling specifically focused on neonatal foals from the Cundinamarca region in Colombia and might not represent the country’s entire equine neonatal population. Likewise, some diagnoses were represented by a limited number of patients; expanding the sample size could improve the accuracy of results related to each pathology and its impact on critically ill neonatal foals.

Many factors can influence health outcomes, and since long-term survival rates in this context are not known, integrating the factors covered in this study with statistics from larger populations of hospitalized neonatal foals may provide valuable insights that will allow for making informed decisions and offering accurate prognoses to owners.

## Conclusions

Periodic analysis of cases in equine neonatology is crucial to improve clinical management in the equine breeding industry. This study highlights the importance of identifying risk factors and optimizing protocols accordingly to enhance neonatal survival rates.
